# Introducing heart rate variability monitoring combined with biomarker screening into a level IV NICU: a prospective implementation study

**DOI:** 10.1007/s00431-022-04534-4

**Published:** 2022-07-04

**Authors:** Şerife Kurul, Nicky van Ackeren, Tom G. Goos, Christian R. B. Ramakers, Jasper V. Been, René F. Kornelisse, Irwin K. M. Reiss, Sinno H. P. Simons, H. Rob Taal

**Affiliations:** 1 Department of Pediatrics, Division Neonatology, Erasmus MC, University Medical Center, Sophia Children’s Hospital, PO Box 2060, 3000 CB Rotterdam, The Netherlands; 2 Department of Biomechanical Engineering, Delft University of Technology, Delft, The Netherlands; 3 Department of Clinical Chemistry, Erasmus MC, University Medical Center, Rotterdam, The Netherlands

**Keywords:** Neonatology, Neonatal sepsis, Heart rate variability monitoring, Interleukin-6, C-reactive protein, Procalcitonin

## Abstract

The aim of this study was to investigate the association between the implementation of a local heart rate variability (HRV) monitoring guideline combined with determination of inflammatory biomarkers and mortality, measures of sepsis severity, frequency of sepsis testing, and antibiotic usage, among very preterm neonates. In January 2018, a guideline was implemented for early detection of late-onset neonatal sepsis using HRV monitoring combined with determination of inflammatory biomarkers. Data on all patients admitted with a gestational age at birth of < 32 weeks were reviewed in the period January 2016–June 2020 (*n* = 1,135; *n* = 515 pre-implementation, *n* = 620 post-implementation). Outcomes of interest were (sepsis-related) mortality, sepsis severity (neonatal sequential organ failure assessment (nSOFA)), sepsis testing, and antibiotic usage. Differences before and after implementation of the guideline were assessed using logistic and linear regression analysis for binary and continuous outcomes respectively. All analyses were adjusted for gestational age and sex. Mortality within 10 days of a sepsis episode occurred in 39 (10.3%) and 34 (7.6%) episodes in the pre- and post-implementation period respectively (*P* = 0.13). The nSOFA course during a sepsis episode was significantly lower in the post-implementation group (*P* = 0.01). We observed significantly more blood tests for determination of inflammatory biomarkers, but no statistically significant difference in number of blood cultures drawn and in antibiotic usage between the two periods.

*Conclusion*: Implementing HRV monitoring with determination of inflammatory biomarkers might help identify patients with sepsis sooner, resulting in reduced sepsis severity, without an increased use of antibiotics or number of blood cultures.

**What is Known:**

*• Heart rate variability (HRV) monitoring might be used as an early warning system to diagnose preterm neonates at risk of developing sepsis*.

*• It has already been shown that HRV monitoring could reduce mortality; however, there are concerns that HRV monitoring alone could lead to higher rates of blood cultures and overuse of antibiotics*.

**What is New:**

*• Implementing HRV monitoring with determination of inflammatory biomarkers might help identify patients with sepsis sooner, resulting in reduced sepsis severity, without an increased use of antibiotics or number of blood cultures*.

## Introduction

Neonatal sepsis is a major health issue, particularly in preterm neonates, where low gestational age, low birth weight, immature immune system, and other compromising factors make it an important cause of morbidity and death [1–5]. Late-onset neonatal sepsis (LONS) is defined as sepsis that occurs after 3 days of life and may be caused by pathogens acquired at delivery or during the course of hospital care [6]. Although in most cases the initiation of LONS is often inconspicuous, the clinical course may be alarmingly fulminant leading to septic shock and death within hours of onset [7, 8]. Therefore, infected neonates must be promptly identified, and appropriate therapy should be started as soon as possible to reduce mortality and morbidity [9, 10]. Consequently, there is a need for early warning signs that could assist clinicians. Inflammatory biomarkers might be used to guide diagnosis in early stages [11]. However, no ideal biomarker has been identified so far and as repeated prophylactic determination of chemical biomarkers is not feasible in preterm neonates, determination of chemical biomarkers is normally performed when symptoms/clinical signs of sepsis are already present. Vital sign analysis may serve as an early warning system to guide the need for biomarker determination early in the sepsis course when no or subtle symptoms are present. Previously, it was observed that sepsis is often associated with decreased heart rate variability (HRV) and transient heart rate decelerations in the hours and days prior to LONS [12, 13]. Heart rate characteristics (HRCs) have been characterized mathematically, and a resulting HRC index, reflecting changes in HRV possibly related to the occurrence of sepsis, can be calculated [14, 15]. It was shown that HRV monitoring significantly reduced mortality in preterm neonates during neonatal intensive care unit (NICU) stay [16]. However, there are concerns that HRV monitoring alone could lead to higher rates of blood cultures and overuse of antibiotics [16–18].

**Fig. 1:**
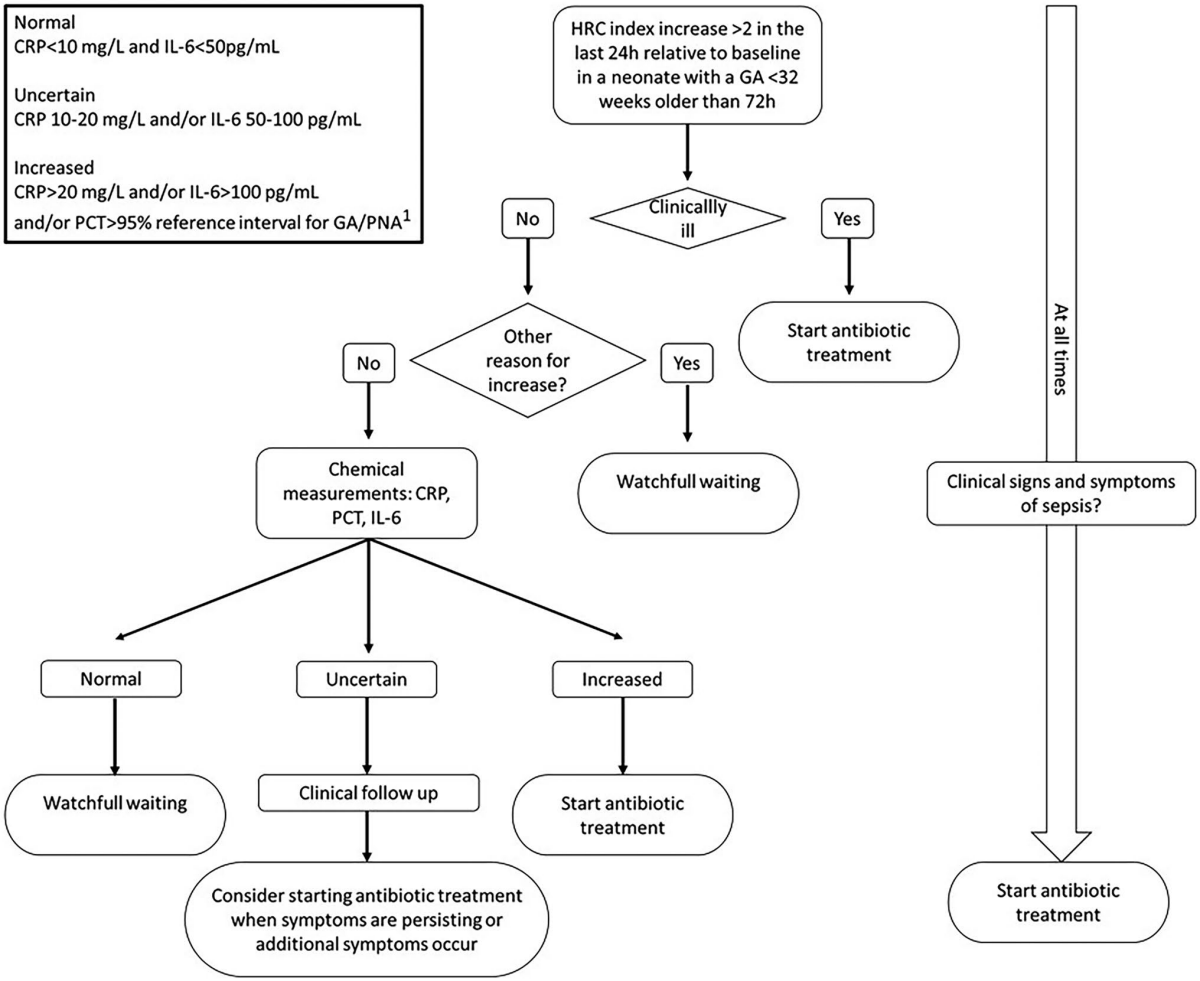
Local HRV monitoring guideline combined with determination of inflammatory biomarkers. ^1^PCT reference interval is based on the results of King [19]. HRC (heart rate characteristic), GA (gestational age)

To address this concern, we implemented as a standard of care a HRV monitoring guideline in which we, based on changes in HRV, determine interleukin-6 (IL-6), procalcitonin (PCT), and C-reactive protein (CRP) to diagnose LONS (see Fig. 1). In apparently septic neonates, antibiotic therapy is always started immediately. The aim of this study was to investigate the association between the implementation of the HRV monitoring guideline in combination with determination of inflammatory biomarkers, on sepsis mortality, sepsis severity, rates of blood cultures, and antibiotic usage.

## Materials and methods

### Study design and population

The study was conducted at the Erasmus MC University Medical Center–Sophia Children’s Hospital Rotterdam, a level-IV NICU. Electronic health record data from January 2016 until December 2017 were reviewed for all neonates with a gestational age below 32 weeks, who got admitted to the NICU. Data from January 2018 until June 2020 were available in a prospectively collected database.

### HRV monitoring guideline

HRV monitoring (HeRO monitor (Medical Predictive Science Corporation (MPSC); Charlottesville, Virginia)) was introduced in January 2018. The HeRO monitor displays a HRC index which is calculated every hour and ranges between 0 and 7. This number reflects the fold increase in risk of sepsis in the next 24 h. Until January 2018, clinical staff was blinded for the HRC index. We developed a local guideline for preterm neonates older than 72 h (Fig. 1). According to this guideline, clinicians can consider determining inflammatory biomarkers when the HRC index is increased more than 2 points, compared to the baseline of the patient. When the inflammatory biomarkers are increased (biomarker levels typically available < 90 min), a blood culture is drawn and antibiotic therapy started (Fig. 1). As changes in HRV can already occur early on in the disease process, before clinical signs are present, we also incorporated inflammatory biomarkers that increase during different phases of the inflammatory cascade in response to sepsis. Cutoff values for the biomarkers were defined as follows: IL-6 levels above 100 pg/mL, PCT levels above the reference interval [19] for gestational age at birth and postnatal age, and CRP levels above 20 mg/L are considered increased and are reason to start with antibiotic therapy. If the patient exhibits clinical signs strongly suggestive of sepsis, antibiotic therapy is always started immediately (see Fig. 1). The pre-implementation period is defined as the period from January 2016 until December 2017 and the post-implementation period from January 2018 until June 2020.

### Patient characteristics

The following patient characteristics were obtained from clinical charts: sex, gestational age, birthweight, postnatal age at moment of blood culture withdrawal. Serum IL-6, PCT (both measured with E801, cobas® 8000 system, Roche Diagnostics, Rotkreuz, Switzerland), and CRP (measured with C502, cobas® 8000 system, Roche Diagnostics, Rotkreuz, Switzerland) levels at moment of sepsis suspicion were queried from the laboratory information system.

### Sepsis definition

The department of Microbiology at the Erasmus MC provided all results of blood cultures, including timing of collection and identified micro-organisms. One patient could provide multiple cases of suspected LONS. LONS diagnosis was established by the criteria defined by the NICHD Neonatal Research Network [20]. An episode of culture-proven LONS was defined as a positive blood culture due to an identified bacterial organism (including coagulase-negative staphylococci), treated with antibiotics for 5 days or more or treated for a shorter duration if death occurred during therapy [20]. Culture negative LONS was defined as (1) C-reactive protein level greater than 10 mg/L within 2 days after blood culture, (2) antibiotic therapy longer than 5 days (or intention to treat longer), and (3) clinical symptoms of sepsis assessed by attending physician. We classified suspected LONS episodes as follows: no sepsis, culture negative sepsis, Gram-positive sepsis, or Gram-negative sepsis. Fungal infections were excluded from analysis.

### Outcome measures

The outcomes of interest were as follows: overall-mortality, 10-day mortality after sepsis suspicion, and measures of sepsis severity; neonatal Sequential Organ Failure Assessment (nSOFA) score [21], need for inotropic therapy, need for mechanical ventilation and severe thrombocytopenia within 3 days of suspicion, the number of blood cultures withdrawn, number of blood withdrawals for inflammatory parameters, and number of antibiotic days for LONS. The nSOFA score consists of respiratory, cardiovascular, and hematological criteria and ranges from 0 to 15 and reflects sepsis severity [21]. The nSOFA parameters were obtained from clinical charts to calculate the nSOFA scores at time points − 24, − 12, − 6, 0, 6, 12, 24, 36, and 48 h relative to blood culture withdrawal. Inotropic support was defined as the start of inotropes after sepsis suspicion within 72 h. Need for mechanical ventilation was defined as the need for intubation within 72 h after suspicion. If the patient was already intubated at moment of sepsis suspicion, this sepsis episode was excluded for analysis for this specific outcome. Severe thrombocytopenia was defined as a platelet count < 50 × 10^9^ cells/L.

### Statistical analysis

Statistical analysis was performed using SPSS (IBM SPSS Statistics for Windows, Version 25.0) and R (R Core Team (2017), Vienna, Austria). Categorical variables were described using absolute numbers and frequencies. Continuous variables were described using medians and interquartile range because of non-normal distributions. Pre-post implementation comparisons were performed using the Mann–Whitney *U* test for continuous data and the chi-square test for categorical data. A logistic regression analysis was used to investigate the difference in binary outcome measures, and a linear regression was used to investigate the difference in continuous outcome measures before and after implementation of the HRV guideline combined with biomarker screening. All analyses were adjusted for gestational age and sex. Additional sensitivity analysis adjusting for underlying trends in treatment strategies was performed. A generalized linear model was built to analyze differences in the nSOFA course during the sepsis course in patients with sepsis. For all tests, a *P*-value of < 0.05 was considered significant.

## Results

### Patient characteristics

In the pre-implementation period, a total of 515 patients (< 32 weeks GA) were admitted to the NICU, in the post-implementation period 620 patients. In the study period, a total of 811 blood cultures in 473 prematurely born neonates (< 32 weeks GA) were withdrawn. Of these episodes, 490 (60.4%) were classified as sepsis with a similar distribution of type of sepsis (see Table 1). Baseline characteristics are summarized in Table 1.

#### Mortality

The overall mortality rate in neonates born with a gestational age < 32 weeks during the study period was 12.0% in the pre-implementation period and 11.3% in the post implementation period (*P* = 0.70). In the pre-implementation period, death within 10 days after blood culture occurred in 39 (10.3%) episodes and in the post-implementation period in 34 (7.6%) episodes (adjusted odds ratio (aOR) 0.69 (95% CI 0.42–1.12) after implementation, *P* = 0.13) (see Table 2). Sensitivity analyses adjusting for underlying trends in treatment strategies slightly reduced effect estimate, however did not change the results materially (see Additional File 1).

**Table 1:** Patient characteristics

***Variable***	***Pre-implementation*** 515 patients	***Post-implementation*** 620 patients	***P-value***
**Sex (male)**	295 (57.3%)	334 (53.9%)	0.14
**Gestational age (weeks + days)**	29 + 0 (27 + 0–30 + 4)	29 + 1 (26 + 6–30 + 4)	0.94
**Birthweight (grams)**	1180 (880–1470)	1173 (870–1510)	0.62
**In-hospital mortality**	62 (12.0%)	70 (11.3%)	0.70
**Age at first blood culture (days)**	8 (6–14)	7.5 (5–12)	0.05
**Number of suspected episodes**			
0 suspected episode	288 (55.9%)	374 (60.3%)	
1 suspected episode	137 (26.6%)	140 (22.6%)	
2 suspected episodes	49 (9.5%)	62 (10.0%)	
> 2 suspected episodes	41 (8.0%)	44 (7.1%)	
**Total number of blood cultures**	377	434	
**Type of sepsis**			
No sepsis	146 (38.7%)	175 (40.3%)	
Culture negative	86 (22.8%)	102 (23.5%)	
Gram-positive	113 (30.0%)	120 (27.7%)	
*Staphylococcus aureus*	25	30	
Coagulase-negative Staphylococci	80	78	
Others	8	12	
Gram-negative	32 (8.5%)	37 (8.5%)	
*Escherichia coli*	17	20	
Others	15	17	
**IL-6 level at onset (pg/mL)**	N/A	113 (44–321)	
**PCT level at onset (ng/mL)**	N/A	0.96 (0.50–2.36)	
**CRP level at onset (mg/L)**	12.0 (2.5–44.8)	11.0 (2.3–36.0)	0.24

Values are medians (25th–75th percentile) or percentages. Differences between pre- and post-implementation groups were assessed using Mann-Whitney *U* tests were used for continuous variables, chi-square test for categorical variables

**Table 2:** Outcome measures

***Variable***	***Pre-implementation*** 377 episodes	***Post-implementation*** 434 episodes	***Odds ratio (95% CI)***	***P-value***
**10-day mortality after blood culture**	39 (10.3%)	34 (7.6%)	0.69 (95% CI 0.42–1.12)	0.13
**Intropic support**	78 (20.7%)	84 (19.4%)	0.94 (95% CI 0.65–1.36)	0.75
**Mechanical ventilation**	*N* = *281* 73 (26.0%)	*N* = *315* 66 (21.0%)	0.71 (95% CI 0.48–1.04)	0.08
**Thrombopenia < 50 × 10**^**9**^** cells/L**	52 (13.8%)	37 (8.5%)	0.57 (95% CI 0.36–0.90)	0.02
			***Effect estimate***	
**Number of bloodcultures drawn**	2 (1–3)	2 (1–3)	−0.04 (95% CI −0.48–0.40)	0.84
**Number of blood tests for inflammatory biomarkers**	1 (1–2)	3 (1–5)	2.1 (95% CI 1.8–2.4)	< 0.001
**Number of antibiotic days**	6 (2–13)	6 (2–14)	−0.114 (95% CI −2.2–2.0)	0.91
**Number of antibiotic days over 1000 hospitalization days**	178.6 (85.5–348.8)	168.1 (84.9–321.9)	−46 (95% CI −93.2–1.3)	0.06

Values are medians (25th–75th percentile) or percentages. Differences between the pre- and post-implementation groups were assessed using logistic regression for binary outcomes and linear regression for continuous outcomes. The effect estimate is the degree of change in the outcome variable between pre- and post-implementation adjusted for gestational age and sex. All analyses are adjusted for gestational age and sex

#### nSOFA score

**Fig. 2:**
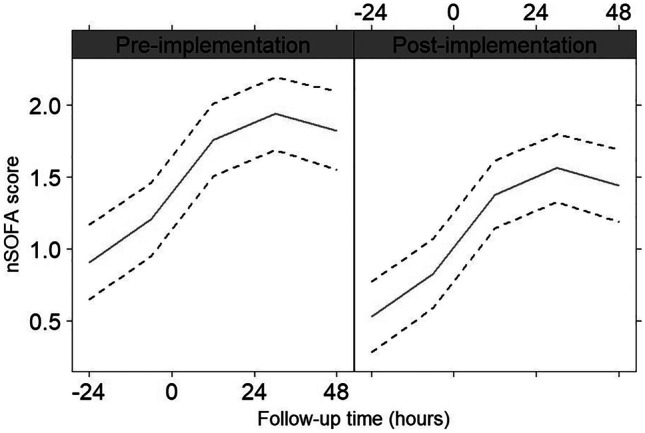
Generalized linear model of the nSOFA scores pre- and post-implementation of HRV monitoring combined with biomarker screening. The black solid line represents the mean of the nSOFA score at different time points relative to the blood culture (*T* = 0). The black dotted lines represent the 95% confidence interval. nSOFA (neonatal sequential organ failure assessment)

Figure 2 shows the nSOFA scores per time point relative to the withdrawal of the blood culture in patients with sepsis. The nSOFA scores both before sepsis suspicion and during the sepsis episode were significantly lower in the post-implementation group (*P* = 0.01).

#### Inotropic support, need for mechanical ventilation, thrombocytopenia

Patients needed inotropic support in 65 episodes (17.2%) and in 74 episodes (17.1%) episodes in the pre-implementation and in the post-implementation period, respectively (aOR 0.94 (95% CI 0.65–1.36) after implementation, *P* = 0.75). Patients needed intubation in 73 episodes (26.0%) and in 66 (21.0%) episodes in the pre-implementation and in the post-implementation period, respectively (aOR 0.71 (95% CI 0.48–1.04) after implementation, *P* = 0.08). Patients had severe thrombocytopenia in 52 episodes (13.8%) and in 37 episodes (8.5%) in the pre-implementation and in the post-implementation period, respectively (21.0%) (aOR 0.57 (95% CI 0.36–0.90) after implementation, *P* = 0.02) (see Table 2).

#### Sepsis testing

The median number of blood cultures drawn per patient in the pre-implementation period was 2 and in the post-implementation period it was 2 (*P* = 0.51). The median number of blood withdrawals for inflammatory biomarkers was 1 in the pre-implementation period and it was 3 in the post-implementation period (*P* < 0.001) (see Table 2).

#### Antibiotic therapy

The median antibiotic days for late-onset sepsis per patient pre-implementation was 6 days and post-implementation it was 6 days (*P* = 0.91). Pre-implementation the number of antibiotic days over hospitalization was 178.6 per 1000 days and post-implementation this was 168.1 per 1000 days (*P* = 0.06) (see Table 2).

## Discussion

Following implementation of a guideline using HRV monitoring combined with determination of inflammatory biomarkers, we observed a significant decrease in the severity of LONS episodes. In line with this, although not statistically significant, 10-day mortality risk following LONS diagnosis also was lower post-implementation. Inherent to the new protocol, there was an increase in number of blood withdrawals to determine inflammatory markers, but the number of blood cultures and antibiotic days were not different.

Our results are in line with the study of Moorman et al. which showed that implementing HRV monitoring led to mortality reduction (hazard ratio of 0.74; 95% CI, 0.57–0.95; *P* = 0.02; number needed to monitor = 23) in a randomized controlled trial including over 3,000 preterm neonates [16]. We also demonstrated that patients in the post-implementation period had a significantly lower mean nSOFA score at onset of sepsis and during the subsequent sepsis course, suggesting that patients were diagnosed earlier and less severely ill. We showed a decreasing trend in need for intubation, need for inotropics, and a statistically significant decrease in severe thrombocytopenia after the implementation of HRV monitoring combined with biomarker screening. In line with our findings, Moorman et al. also showed a trend towards increased ventilator-free days alive with HRV monitoring [16].

Our study did not show an increase in the numbers of blood cultures drawn and in antibiotic usage. In the randomized trial of Moorman et al., neonates exposed to HRV monitoring had a 10% increase in number of blood cultures withdrawn compared to patients without monitoring, although this was not statistically significant [16]. Furthermore, it was shown in a previous study that patients who were exposed to HRV monitoring received on average 3 days more antibiotics than patients without HRV monitoring [17]. Antibiotic overuse could lead to adverse effects, such as necrotizing enterocolitis [22], altered gut colonization [23], and increased *Candida* colonization [24]. On top of that, antibiotic overuse could promote bacterial antibiotic resistance [25]. Hence, overuse of antibiotics should be avoided.

During HRV monitoring, the HRC index can be increased without being related to sepsis, in situations of respiratory insufficiency or severe apnea and/or bradycardias [26]. In a cohort of 2384 patients, it was found that in more than 90% of the times that the HRC index was increased, it was not related to sepsis [27]. Adding inflammatory biomarker screening at the moment of an increase in HRC index could assist in decisions about evaluation for sepsis [26]. Therefore, we incorporated inflammatory biomarkers in our HRV monitoring guideline as diagnostic approach, which could reduce false positive rates of HRV monitoring alone (leading to an increased antibiotic use and blood cultures withdrawn). Our study consequently showed a significantly increased number of blood withdrawals to measure inflammatory biomarkers. One way to reduce blood drawn for determining inflammatory biomarkers is to increase our understanding of the HRV monitor. As an increase in HRC score can be due to various reasons, also unrelated to sepsis [28, 29], understanding and training clinical staff in interpreting changes in HRV will help identify true sepsis-related changes in HRC scores [28], leading to a decreased number of blood withdrawals for inflammatory biomarkers.

In the past years, other trends in treatment strategies of LONS may underlie the differences in the pre- and post-implementation periods, with regard to clinical outcomes. For example, we introduced pentoxifylline (PTX) treatment. PTX is a phosphodiesterase inhibitor, originally registered for intermittent claudication in adults [28], which suppresses the production of TNFα and other inflammatory cytokines and prevents their subsequent effects [30, 31]. From January 2020 onwards, we start PTX in cases of suspected sepsis when IL-6 levels are above 500 pg/mL and/or CRP levels are above 50 mg/mL at onset [32]. Therefore, we performed a sensitivity analysis adjusting for underlying trends in treatment strategies. This analysis showed that the effect estimate of the introduction of the HRV guideline was slightly reduced. This suggests that underlying trends might partially explain the effect on sepsis-related mortality. Excluding the last 6 months (introduction of PTX treatment) of the post-implementation period did not change the effect estimate materially (data not shown). Aside from introducing PTX, we did not implement other changes in the management of LONS (e.g., empirical antibiotic therapy) during the study period that could explain the differences in the pre- and post-implementation periods. Regarding sepsis severity, we have shown that patients were less severely ill at onset of sepsis suspicion. As we did not change monitoring strategies apart from HRV monitoring, the effect is likely due to implementation of HRV monitoring.

Additionally, our PTX protocol is an example on how to use an early warning system followed by determination of inflammatory biomarkers that give information about the subsequent disease course, for practicing pro-active personalized treatment [32, 33].

Our study has some limitations. We performed a cohort study including all patients with a gestational age < 32 weeks admitted to our hospital. This type of study does not allow for a concurrent control group, limiting our ability to attribute causation. We obtained many variables and outcome data from clinical charts, which may lead to misclassification due to mistakes in data entry. If misclassification would occur, we would expect this to be random decreasing precision of the effect estimates. We tried to limit missing data and misclassification by double data entry and using automated registries for blood cultures from the department of microbiology.

Also, although the current study reports on one of the largest cohorts with HRV monitoring, the sample size is still relatively small. The number needed to monitor for saving one life, reported by Moorman et al., is 23 patients [16]. The non-statistically significant trend towards a lower sepsis-related mortality is likely to reflect the limited sample size for this outcome. Future research should focus on validating our findings in independent and larger populations. Causality could be assessed by a randomized controlled trial or a quasi-experimental study with several NICUs with different starting time of HRV implementation. This could help to implement the HRV monitor on a larger scale in the neonatal intensive care to enable timely detection of LONS.

In conclusion, our findings show a trend towards lower 10-day mortality after sepsis suspicion and a significant reduction of sepsis severity after implementation of HRV monitoring combined with determination of inflammatory biomarkers. We observed no increase in antibiotic usage and the frequency of sepsis testing. However, this guideline logically was associated with an increase in blood withdrawals to determine inflammatory biomarkers. Increasing our understanding in HRV monitoring could potentially help reduce this. Implementing HRV monitoring with determination of inflammatory biomarkers might help identify patients with sepsis sooner, resulting in reduced sepsis severity, without an increased risk of increased antibiotic usage and sepsis testing.

## Data Availability

The data that support the findings of this study are available on request from the corresponding author. The data are not publicly available due to privacy or ethical restrictions.

## Abbreviations

CRP: C-reactive protein
GA: Gestational age
HRV: Heart rate variability
HRC: Hear rate characteristic
IL-6: Interleukin-6
LONS: Late-onset neonatal sepsis
NICU: Neonatal intensive care unit
nSOFA: Neonatal sequential organ failure assessment
PCT: Procalcitonin
PTX: Pentoxifylline

## Code availability

The code that support the findings of this study is available on request from the corresponding author (SPSS (IBM SPSS Statistics for Windows, Version 25.0) and R (R Core Team (2017), Vienna, Austria)).
